# Reactive transport modeling of organic carbon degradation in marine methane hydrate systems

**DOI:** 10.1038/s41598-024-52957-w

**Published:** 2024-02-03

**Authors:** Li Wei, Alberto Malinverno, Frederick Colwell, David S. Goldberg

**Affiliations:** 1 Lamont-Doherty Earth Observatory, Columbia University, Palisades, NY 10964 USA; 2 College of Earth, Ocean, and Atmospheric Sciences, Oregon State University, Corvallis, OR 97331 USA

**Keywords:** Biogeochemistry, Ocean sciences

## Abstract

Natural methane hydrate has often been observed in sand layers that contain no particulate organic carbon (POC), but are surrounded by organic-rich, fine-grained marine muds. In this paper, we develop a reactive transport model (RTM) of a microbially-mediated set of POC degradation reactions, including hydrolysis of POC driven by extracellular enzymes, fermentation of the resulting high-molecular weight dissolved organic carbon (HMW-DOC), and methanogenesis that consumes low-molecular weight dissolved organic carbon (LMW-DOC). These processes are mediated by two groups of microbes, fermenters and methanogens that are heterogeneously distributed in different lithologies, with the largest numbers of microbes in the large pores of coarse-grained layers. We find that the RTM can reproduce methane hydrate occurrences observed in two different geological environments, at Walker Ridge Site 313-H (Gulf of Mexico) and IODP Site U1325 (Cascadia Margin). We also find that microbes can degrade POC even if they are physically separated, as extracellular enzymes and DOC can diffuse away from where they are produced by microbes. Microbial activity is highest at relatively early times after burial at shallow depths and near lithological boundaries, where concentration gradients transport solutes to intervals that contain the most microbes.

## Introduction

Natural methane hydrate has been widely observed in marine continental margin sediments^1,2^. The methane in the hydrate is predominantly produced by microbially-mediated degradation of sedimentary particulate organic carbon (POC)^3–6^. However, methane hydrate is preferentially found in coarse-grained sediment layers (sand or coarse silt) where POC is generally absent, whereas adjacent organic-rich fine-grained muds contain little to no hydrate. Reactive transport models (RTMs) have been applied to explore the formation mechanism of microbial methane and the resulting hydrate occurrence in marine sediments. In these models, the complex process of POC degradation has been generally represented as a one-step conversion of POC to methane. Microbial methane generated in fine-grained muds is then transported by diffusion or fluid flow into adjacent sand layers, where hydrate formation is not hindered by small pore sizes and hydrates can accumulate^7–9^.

Previous RTMs generally do not account for the number and distribution of microbial cells in sediments. There is evidence that subsurface microbial cells are more concentrated in coarse-grained sediments, whose larger pores permit microbial activity and survival^10–12^. For example, the number of microbial cells in fine-grained muds decreases from ~ 10^9^ per cm^3^ of sediment near the seafloor to ~ 10^6^ at depth where pore compaction reaches steady state^12^. In contrast, the number of cells remained nearly constant (~ 10^9^ per cm^3^) in coarse-grained sediments that experienced only minor compaction. While sand layers contain little organic matter yet concentrated microbes, it is not clear how do these microbes get access to the solid organic matter in the surrounding muds and eventually form methane hydrate in the sand.

**Figure 1:**
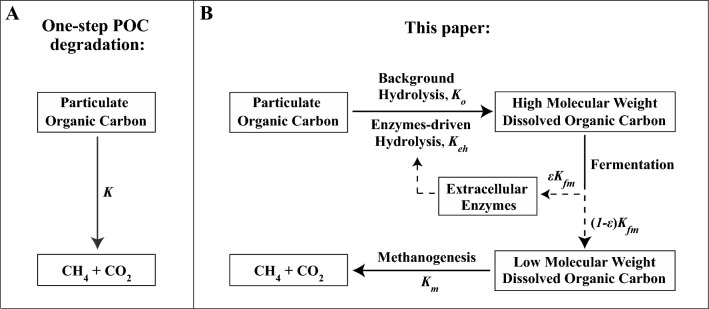
Schematics of the microbially-mediated degradation of particulate organic carbon (POC) in marine methane hydrate system. (**A**) In a simplified one-step process, POC is converted to methane and CO_2_ with a single rate constant $K$. (**B**) In this paper, POC is hydrolyzed with a reaction rate constant ${K}_{eh}$ driven by extracellular enzymes and a small background rate constant ${K}_{o}$. The produced high-molecular weight dissolved organic carbon (HMW-DOC) is fermented to generate low-molecular weight DOC (LMW-DOC) with a rate constant $(1-\varepsilon ){K}_{fm}$ and small amounts of extracellular enzymes at a rate constant $\varepsilon {K}_{fm}$ ($\varepsilon \ll 1$). LMW-DOC is then metabolized at a rate constant ${K}_{m}$ to produce terminal end products methane and CO_2_.

The complex POC degradation process in marine sediments is a sequence of microbially-mediated reactions that break down organic molecules to sufficiently small sizes to pass through various microbial cells^13–19^. POC is initially hydrolyzed to generate dissolved organic carbon (DOC) intermediates. The DOC intermediates are then sequentially fermented to progressively decreasing molecular weights, which are eventually utilized in terminal respiratory process such as methanogenesis^13,14^. Figure 1 shows the conceptual description of this complex POC degradation process in comparison with the simplified one-step POC conversion used in previous methane hydrate RTMs (Fig. 1A). In the more complex POC degradation process (Fig. 1B), POC is hydrolyzed at a background rate constant ${K}_{o}$ and an extracellular enzyme-driven rate constant ${K}_{eh}$. The resulting high molecular weight DOC (HMW-DOC) is fermented to produce low molecular weight DOC (LMW-DOC) at a rate constant $(1-\varepsilon ){K}_{fm}$ and extracellular enzymes at a rate constant ${\varepsilon K}_{fm}$. A small constant $\varepsilon \ll 1$ is applied to represent only a small fraction of HMW-DOC is required to generate extracellular enzymes. Finally, LMW-DOC is metabolized at a rate constant ${K}_{m}$, generating end products of methane and CO_2_.

In this work, we develop an RTM for the more complex description of POC degradation process that forms microbial methane and hydrate in a sand layer surrounded with organic-rich mud. Two types of microbes are active in the POC degradation process: fermenting microbes that consume HMW-DOC compounds and produce LMW-DOC and extracellular enzymes; and methanogens that metabolize LMW-DOC and produce methane and CO_2_. Moreover, the RTM accounts for the different number of microbial cells in different lithologies, where microbes are significantly more abundant in the coarse-grained sand layer than in the muds. This is an important first step in improving RTMs of microbial processes in methane hydrate-bearing sediments, moving beyond “microbial models without microbes”^20^. The RTM is applied in two different continental margin settings where methane hydrate was observed in sand layers of different thicknesses. The first location is at Site Walker Ridge 313-H (WR313-H) in the passive margin of the Gulf of Mexico, where a 3 m-thick hydrate-bearing sand was observed bounded by hydrate-free muds and has been previously modeled^21–23^. At the second location, hydrate was observed in numerous 5 cm-thick, thin sands bounded by thicker fine-grained muds at IODP Site U1325 in the active Cascadia margin^8^. The RTM predictions for DOC and methane hydrate are then compared to the detailed observations made at the two Sites.

## Modeling the POC degradation process

### Mass balance equations and finite difference solutions

We represent the overall process of POC degradation (Fig. 1) with a quantitative model. This involves balancing the mass of carbon among six carbon-bearing compounds: POC, HMW-DOC, LMW-DOC, extracellular enzymes, methane, and methane hydrate. The conservation of carbon mass for each compound is described by a set of coupled, mass-balanced, partial differential equations (referring to Supplementary Eqs. (5), (6), (7), (9), (19), (21)). To solve these mass balance equations, we apply a finite difference implicit method^24^ within a Lagrangian framework. In this framework, the depth coordinate is fixed to the top of a sediment interval originating at the seafloor and gradually undergoing burial.

The finite difference solutions are computed using a three-fractional step scheme that achieves second-order accuracy^24^. The first fractional step accounts for the diffusion of solutes (HMW-DOC, LMW-DOC, extracellular enzymes, and methane) over a half time step. The second fractional step incorporates reaction terms that undergo changes over a full time step. These reaction terms include POC hydrolysis, leading to the production of HMW-DOC; HMW-DOC fermentation, resulting in the generation of LMW-DOC and extracellular enzymes; the decay of extracellular enzymes; LMW-DOC methanogenesis, yielding generation of methane; and the formation/dissolution of methane hydrate. The third fractional step repeats the procedure in the first fractional step, accounting for solute diffusion over another half time step. Detailed explanation of the finite difference calculations is described in the Supplement (Sect. 1.3).

### Initial and boundary conditions

The sediment interval in the RTM comprises a thin sand layer positioned at the domain’s center, characterized by no POC content and a high concentration of microbes. The sand layer is bounded by thicker fine-grained mud intervals above and below, which contain a small amount of POC and fewer microbes. The selection of microbe quantities in the two lithologies and their temporal variation with burial is informed by prior data compilation and modeling efforts estimating 10^9^ cells/cm^3^ in the sand and 10^6^ cells/cm^3^ in the mud^12^. At the outset, the concentrations of various solutes in the pore fluid (defined as the molar mass of carbon per volume of pore fluid in mM) including DOC, extracellular enzymes, methane, and the saturation of methane hydrate (defined as the volume fraction of hydrate in the pore), are set to zero. Throughout the burial process, the sediment interval is treated as a closed system with no carbon flux entering or exiting the interval and all reactions occurring within the modeled domain. To enforce this closed system, zero-gradient Neumann boundary conditions (BCs) are imposed at the top and bottom of the sediment interval. Further details on the implementation of boundary conditions are in the Supplement (Sect. 1.3).

### Modeling assumptions

The RTM employs a constant assumed porosity in the two lithologies as sediment undergoes burial and over time, which leads to no advective pore water flow between mud and sand layers. This is a simplification, but it helps for the purpose of our study as we want to test whether methane hydrate can be formed in the sand even if there is no transport of methane by advective pore water flow. Under this assumption, sediment compaction is neglected in both mud and sand, and the solute in pore fluid along with the solid phases are buried at the same sedimentation rate. In the Lagrangian framework, the assumption sets to zero all advection terms in the mass balance equations (Supplementary Eqs. (5), (6), (7), (9), (19), (21)). It is important to note the difference between sediment porosity and pore size distribution: we assume similar porosities for mud and sand, but the pore sizes will be significantly smaller in the mud, limiting microbial population. The effect of small pore sizes in mud also inhibits hydrate formation due to the higher solubility of methane. Conversely, sediments with larger pores, such as coarse-grained sand layers, exhibit lower local solubility of methane, favoring the accumulation of hydrate^8,9,25^.

The RTM excludes the sulfate reduction process and is specifically designed to operate below the sulfate-methane transition zone, for the purpose of testing the POC degradation process depicted in Fig. 1B. Additionally, the RTM operates under the assumption of local thermodynamic equilibrium. This means that once the methane concentration surpasses (or falls below) its solubility limit, hydrate formation (or dissolution) occurs rapidly at a kinetic rate significantly faster than that of all other reactions. To account for the impact of varying microbial populations in different lithologies, the reaction rate constants for microbially-driven processes like fermentation and methanogenesis (denoted as ${K}_{fm}$ and ${K}_{m}$) are expressed as rates per microbial cell. These rates are then multiplied by the number of microbes in the source/sink terms within the mass balance equations (refer to Supplementary Eqs. (6), (7), (9)).

The RTM posits that the initiation of the POC degradation process involves a background hydrolysis propelled by pore water at an exceedingly slow background rate constant denoted as ${K}_{o}$. This assumption is applied to kickstart the POC degradation process given that the initial concentrations of DOC and extracellular enzymes in the pore fluid are both zero. The rate constant of background hydrolysis, ${K}_{o}$, is defined as ${K}_{o}=\beta {E}_{0}{K}_{eh}$, where ${K}_{eh}$ represents the POC hydrolysis rate constant driven by extracellular enzymes, ${E}_{0}$ is a reference extracellular enzyme concentration (set as 0.5 mM in this case, determined from the RTM’s average enzyme concentration), and $\beta$ is a small constant much less than 1(here specified as $\beta =0.01$). To further streamline the RTM, an additional assumption is made that fermenting microbes consume HMW-DOC at a rate constant similar to the rate at which methanogens consume LMW-DOC. Consequently, the rate constant ${K}_{fm}$ is equal to the rate constant ${K}_{m}$, providing a simplification to the model.

In the RTM, methane originates exclusively from the conversion of LMW-DOC. Other processes, such as CO_2_ reduction or hydrogen production from fermentation, are not considered here. The outcomes of the RTM are notably sensitive to three key microbial reaction rate constants. First, the POC hydrolysis rate constant, ${K}_{eh}$, governs how extracellular enzymes enhance POC hydrolysis to generate HMW-DOC. Secondly, the fermentation rate constant, ${K}_{fm}$, dictates the pace at which fermenting microbes consume HMW-DOC to produce LMW-DOC and extracellular enzymes. Lastly, the methanogenesis rate constant, ${K}_{m}$, determines the rate at which methanogens consume LMW-DOC to produce methane, and it is assumed to be equal to ${K}_{fm}$. As these reaction rate constants are not independently known, we compare the RTM predictions with local observations to narrow down the range of reference values for each of the rate constants, ${K}_{eh}$ and ${K}_{fm}$ (or ${K}_{m}$).

We assume that the extracellular enzymes released in sediment pore water decay at a rate constant, ${K}_{ed}={\text{log}}(2)/\lambda$, where $\lambda$ represents the half-life time of the extracellular enzymes. Unfortunately, the lifetimes of extracellular enzymes in marine sediments are poorly constrained^26–29^. Available evidence suggests that these extracellular enzymes in marine sediments must persist over long timescales, given that microbial metabolism is extremely slow in these environments^26,30–33^. Estimates of extracellular enzymes lifetime range from 0.4 to 12,000 years, based on different enzymes specific activity rates^26^. In this study, we experiment with the RTM using various values of $\lambda$ and observe that the POC degradation and methane generation are not noticeable when $\lambda$ is less than 5 kyr. Conversely, when $\lambda$ exceeds 15 kyr, we observe that the POC degradation occurs too rapidly, resulting in high saturations of methane hydrate even in shallow sediments below the seafloor. Consequently, in our model we set the half-life of extracellular enzymes, $\lambda$, to be 10 kyr. This is a pragmatic choice, implemented for the purpose of testing how concentrated microbes in sand access POC in mud to accumulate methane hydrate.

## Hydrate occurrence in two different geological settings

### Walker Ridge Site 313-H, Gulf of Mexico

**Figure 2:**
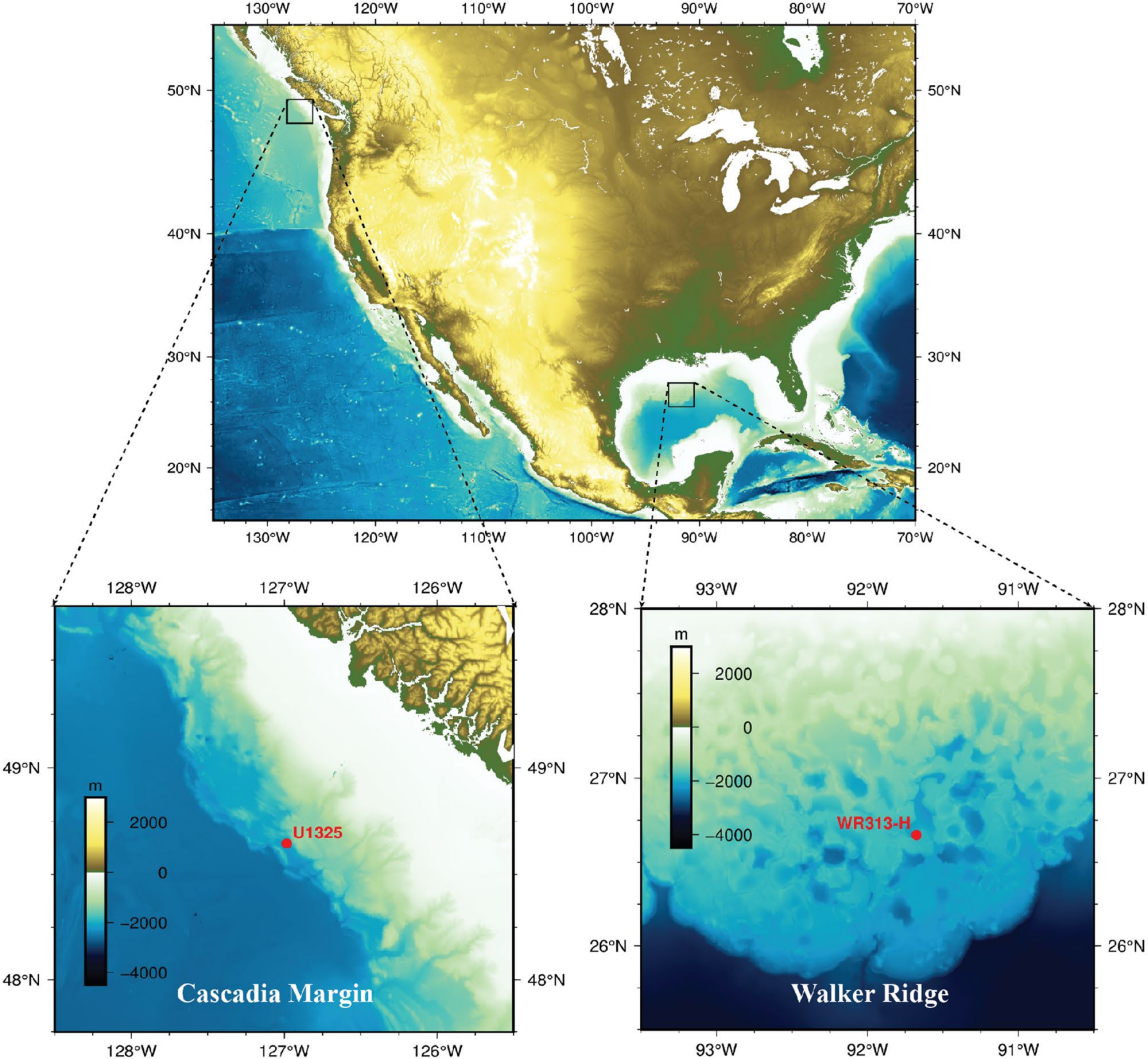
Location map of IODP Site U1325 (Cascadia Margin) and Site WR313-H (Walker Ridge). Color images show water depth in meters.

Site Walker Ridge 313-H (WR 313-H) is in the Terrebonne Basin, northern Gulf of Mexico at a water depth of about 2 km (Fig. 2). During Gas Hydrate Joint Industry Project (JIP) Leg II, logging-while-drilling (LWD) measurements were collected from Site WR 313-H targeting the hydrate reservoirs near the base of gas hydrate stability zone (BGHSZ) at 900 mbsf^34,35^. Methane hydrate was also found in several shallower, coarse-grained intervals, including a 3 m-thick sand near 290 mbsf^34^. The 3-m sand unit is not clearly connected to the deeper methane sources beneath the BGHSZ^36^ and has been used to test one-dimensional microbial POC conversion and methanogenesis models^21–23^. The assumed constant sedimentation rate at WR 313-H is 1 mm/year for the top 300 mbsf, based on sparse biostratigraphic data^21^. As no core data were collected at WR 313-H for organic carbon measurements, it is assumed that only a small amount of POC (0.5 wt%) is available for microbial methane generation^8,37^.

**Figure 3:**
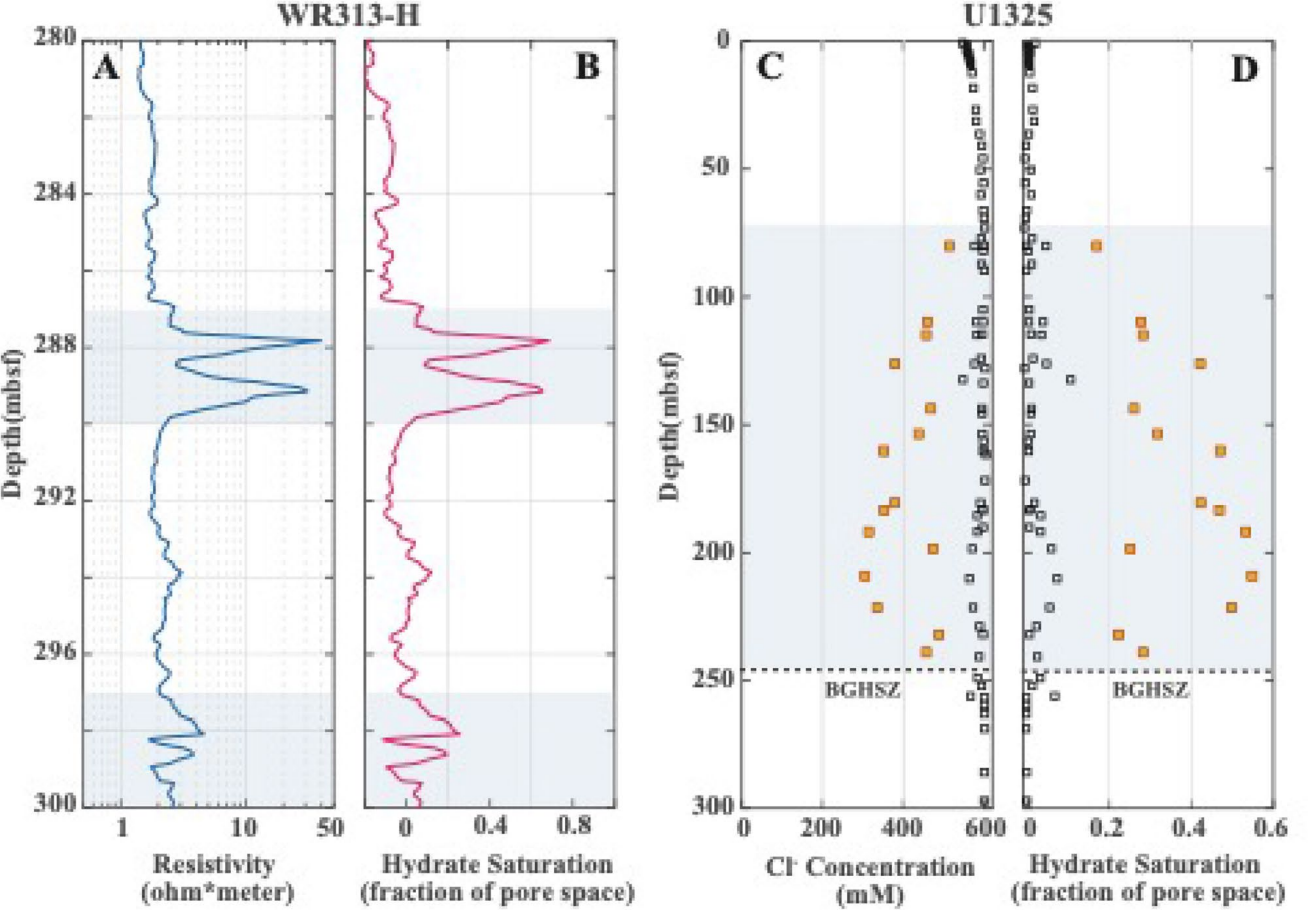
(**A**) Downhole ring resistivity logs at Site WR313-H. (**B**) Hydrate saturation in sediments at Site WR313-H. The depth interval highlighted in gray indicates the presence of methane hydrates in a high resistivity anomaly, including a 3 m-thick sand layer (287–290 mbsf) and hydrate-filling fractures in fine-grained mud (297–300 mbsf). (**C**) Chlorinity in pore waters from mud (black squares) and thin sand layers (orange squares) at IODP Site U1325. (**D**) Hydrate saturation computed from chlorinity data at Site U1325. The depth interval highlighted in gray indicates the presence of methane hydrates based on the low chlorinity anomaly. The top occurrence of hydrate is at ~ 73 mbsf and the bottom of hydrate occurrence is at the base of gas hydrate stability (BGHSZ) at 248 mbsf.

In Fig. 3A, the presence of methane hydrate in the 3-m sand unit at 290 mbsf is indicated by an elevated LWD measured resistivity. The hydrate saturation, defined as the volume fraction of pore space, is ${S}_{H}$ = 0.45 on average predicted from resistivity in the sand layer^21^ (Fig. 3B). The maximum hydrate concentrations occur near the sand bed boundaries. The hydrate-bearing sand is sandwiched by hydrate-free-zones (HFZs) with 10-m (top) and 3-m (bottom) thick fine-grained muds, which are then surrounded by fine-grained sediments with hydrate-filling fractures^21^. Models have shown that similar mud-sand-mud sandwich patterns can be observed where methane generated from fine-grained, organic-rich mud diffuses into the sand layer to form hydrate^8,9,21^. The 3-m sand is also a target unit in the recent Gulf of Mexico Deepwater Hydrate Coring Expedition (GOM^2^-2), where in-situ coring, geochemistry and microbiological data have been acquired to assess the role of microbial methanogenesis in hydrate formation.

### IODP Site U1325, Cascadia margin

Site U1325 was drilled during IODP Expedition 311 in a slope basin of the Cascadia accretionary wedge at a water depth of about 2 km (Fig. 2). Downhole logging and pore water chemistry analyses show gas hydrate is present from a shallowest occurrence at 73 mbsf down to the BGHSZ at 245 mbsf^8,38^. ${S}_{H}$ predicted from detailed pore water chlorinity measurements (Fig. 3C) is estimated to be 0.2 to 0.6 of pore space in thin sand layers (typically < 5 to 10 cm)^39^ (Fig. 3D). The thin hydrate-bearing sand layers are separated by much thicker fine-grained mud intervals that are 2.5 m on average and contain little or no gas hydrate^8^.

The organic carbon content in sediments (defined as mass fraction of sediment grains) at Site U1325 decreases with depth from about 1 wt% near the seafloor to about 0.5 wt% at depths below the BGHSZ^40^. Therefore, we assume that the labile POC available for microbial methane generation is 0.5 wt%. A constant sedimentation rate is estimated to be 0.19 mm/year, based on the 1 Ma age of diatoms collected from cores at 196 mbsf^41^. Site U1325 is markedly different from Site WR 313-H in the Gulf of Mexico, having a lower sedimentation rate, much thinner sand layers, and geological setting on an active continental margin.

## Modeling results at Sites WR 313-H and U1325

### Estimation of reaction rate constants at Site WR 313-H

**Figure 4:**
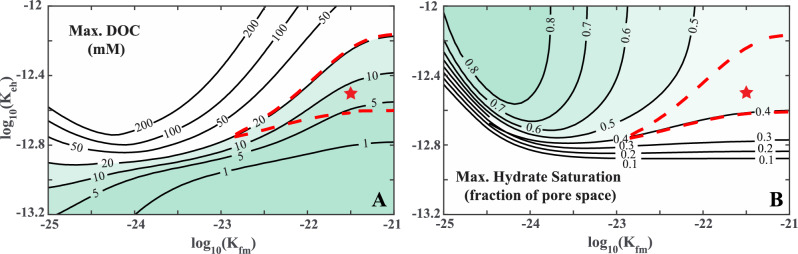
(**A**) Computed maximum dissolved organic carbon (DOC) concentration and (**B**) Maximum hydrate saturation in a 3 m-thick sand at Site WR313-H as a function of microbial reaction rate parameters (enzyme-driven hydrolysis rate constant ${K}_{eh}$ and fermentation rate constant ${K}_{fm}$). The red dashed line marks the region where maximum DOC is below 20 mM and hydrate saturation is greater than 0.4, which is consistent with the field observations at Site WR313-H. The star symbol in the center of the region indicates the chosen representative values of ${K}_{eh}$ and ${K}_{fm}$ at this Site. The right-hand side of the region is open, showing that it can be extended in that direction with an increased fermentation rate constant.

The RTM results are significantly affected by the microbial reaction rate constants, specifically the POC hydrolysis rate constant (${K}_{eh}$) driven by extracellular enzymes and the HMW-DOC fermentation rate constant (${K}_{fm}$), which is assumed equal to the methanogenesis rate constant (${K}_{m}$). To derive reference values for these rate constants, we compute RTM predictions across a range of values for ${K}_{eh}$ and ${K}_{fm}$(or ${K}_{m}$) and constrain them based on two criteria: (1) the concentration of DOC in pore water, measured in settings akin to those of Site WR 313-H (Fig. 4A), and (2) the observed hydrate saturation (${S}_{H}$) in the 3 m-thick sand layer (Fig. 4B).

In addressing constraint (1), we analyzed 812 pore water chemistry datasets obtained from Scientific Ocean Drilling Sites across 26 diverse locations (refer to the summary in Supplement Sect. 3, Table S2). Our analysis revealed that the concentration of DOC in pore water is generally below 20 mM in continental margin marine sediments^6,14,42–49^. There are a few exceptions, such as Blake Ridge, where the maximum DOC concentration reaches 93.4 mM^50^, and the Peru Margin, where it peaks at 23.58 mM^49^. As for constraint (2), we require that RTM results must yield a hydrate saturation (${S}_{H}$) greater than 0.4 of pore space in the 3 m-thick sand layer to match observations from LWD measurements made at Site WR 313-H. The range of reaction rate constants satisfying both constraints (1) and (2) is shown in Fig. 4. Subsequently, we selected representative estimates at WR 313-H, with ${K}_{eh}$ = 10^–12.6^ mM^–1^ s^–1^ and ${K}_{fm}$ (or ${K}_{m}$) = 10^–21.5^ s^–1^, denoted by the star symbol in Fig. 4.

### RTM results at Site WR 313-H

**Figure 5:**
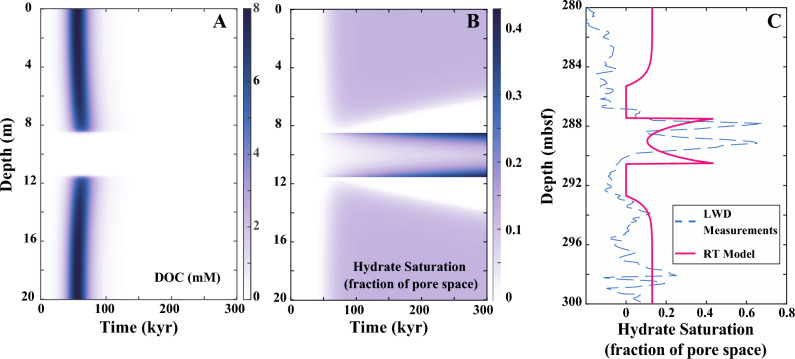
Results of reactive transport modeling (RTM) at Site WR313-H. (**A**) Modeled dissolved organic carbon (DOC) concentration, and (**B**) Modeled hydrate saturation in a 20 m-thick mud-sand sediment interval at WR 313-H as a function of time. A 3 m-thick sand is at the center of the interval surrounded by fine-grained muds. (**C**) Modeled hydrate saturation when the 3 m-thick sand layer is buried at 287–290 mbsf after ~ 300 kyr (red line) compared to hydrate saturation estimated from observed electrical resistivities (dashed blue line).

Figure 5 illustrates the RTM results for the evolution of DOC concentration and hydrate formation resulting from the POC degradation process. This is based on the application of the two representative rate values determined in “Estimation of reaction rate constants at Site WR 313-H” section. The pore water concentration of DOC is computed as the sum of HMW-DOC and LMW-DOC concentrations. At the initial time, the DOC concentration is uniformly zero throughout the sediment interval (Fig. 5A). Subsequently, DOC becomes observable in the fine-grained mud after approximately 30 kyr, reaching a peak value of 8 mM around 60 kyr. Following this peak, the DOC concentration decreases to below 2 mM after about 75 kyr, and nearly diminishes to zero by the 100 kyr mark. Throughout the entire modeling duration, DOC is exclusively observed in the mud intervals, consistently absent in the 3-m sand layer.

In Fig. 5B, methane hydrate starts forming in the sediment interval after approximately 50 kyr. With sediment buried to deeper depth, the hydrate saturation within the sand layer rises, with the highest saturations occurring near the top and the base of the layer. Two HFZs develop immediately above and below the sand layer, and the thickness of these HFZs increases over the modeling period. By the time the sediment interval is buried to 300 mbsf at 300 kyr, the hydrate saturation reaches a maximum of 0.43 in the sand layer, and each HFZ has a thickness of 2 m.

Figure 5C compares RTM predicted hydrate occurrences in the sediment interval between 287 and 290 mbsf with those observed from LWD measurements. The RTM predicts hydrate saturation ranging from ${S}_{H}$ = 0.1 to 0.4 of the pore space in the sand layer, with the maximum value occurring at the bed boundaries. In the adjacent fine-grained muds, a minimal amount of hydrate (${S}_{H}$ = 0.15) is observed above and below the 2 m thick HFZs. A similar pattern is shown in the hydrate estimations from LWD, with hydrate saturation varying from ${S}_{H}$ = 0.1 to 0.7 in the sand layer. The HFZs surrounding it are over 3-m thick, generally exhibiting a small amount of hydrate saturation (${S}_{H}$ $\le$ 0.1) in the fine-grained muds.

### Estimation of reaction rate constants at Site U1325

**Figure 6:**
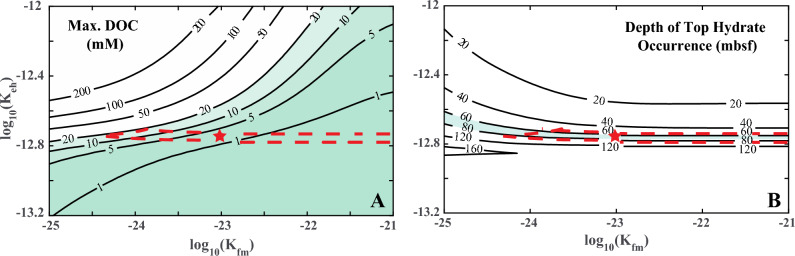
(**A**) Computed maximum dissolved organic carbon (DOC) concentration in sediments and (**B**) Depth of top hydrate occurrence within the GHSZ at Site U1325 as a function of microbial reaction rate parameters (enzymes-driven hydrolysis rate constant ${K}_{eh}$ and fermentation rate constant ${K}_{fm}$). The red dashed line marks the region where maximum DOC is below 20 mM and the top of the hydrate-bearing interval occurs between 60 and 80 mbsf, which is consistent with the observations at Site U1325. The star symbol in the center of the region indicates the chosen representative values of ${K}_{eh}$ and ${K}_{fm}$ at this Site. The right-hand side of the region is open, showing that it can be extended in that direction with an increased fermentation rate constant.

We apply an approach similar to that used for Site WR 313-H to derive reference values for the reaction rate constants ${K}_{eh}$ and ${K}_{fm}$ (or ${K}_{m}$) at Site U1325 (Fig. 6). As detailed in “Estimation of reaction rate constants at Site WR 313-H” section, the first constraint ensures the maximum modeled DOC concentration in pore water does not exceed 20 mM. The hydrate saturations observed in the thin sand layers of Site U1325 are variable. Near the top of the depth interval, hydrate occupies approximately ${S}_{H}$ ~ 0.2 of pore space and increases to approximately ${S}_{H}$ ~ 0.5 near 240 mbsf immediately above the BGHSZ (Fig. 3D). Therefore, the second constraint on the reaction rate sets the shallowest depth at 73 mbsf, where ${S}_{H}$ reaches 0.2, mirroring observations at Site U1325. Figure 6 illustrates the range of reaction rate constants considering these two constraints. We have selected values of ${K}_{eh}$ = 10^–12.7^ mM^–1^ s^–1^ and ${K}_{fm}$ (or ${K}_{m}$) = 10^–23^ s^–1^ as representative estimates at Site U1325, denoted by the star symbol in Fig. 6A,B.

### RTM results at Site U1325

**Figure 7:**
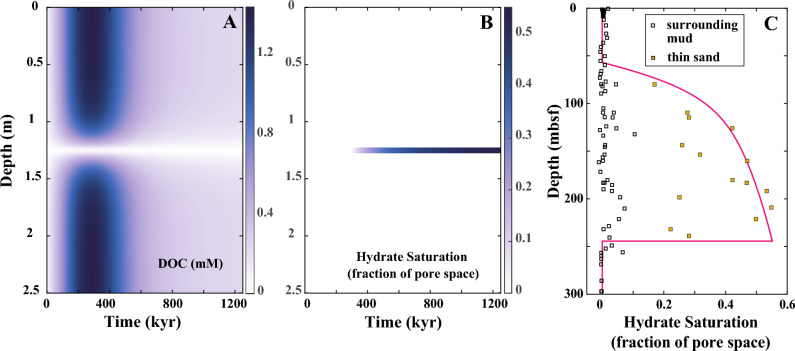
Results of reactive-transport modeling (RTM) at Site U1325. (**A**) Modeled dissolved organic carbon (DOC) concentration, and (**B**) Modeled hydrate saturation in a 2.5 m-thick mud-sand sediment interval at U1325 as a function of time. A 5 cm-thick thin sand is at the center of the interval surrounded by fine-grained muds. (**C**) Modeled hydrate saturation during burial of the 5 cm sand (red line) compared to estimates from observed pore water chlorinity (squares). The model predictions are set to match the overall hydrate saturations observed in thin sand layers (orange squares).

Figure 7 presents the RTM outcomes for the evolution of DOC concentration and hydrate formation, applying the representative rate constant values established in “Estimation of reaction rate constants at Site U1325” section. According to the RTM results, DOC begins accumulating almost immediately after the initial time and reaches a peak of 1.4 mM after approximately 300 kyr (Fig. 7A). After 400 kyr, DOC concentrations decrease to below 1 mM. Similar to the RTM observations at Site WR 313-H, DOC is exclusively observed in the surrounding muds and is absent in the sand layer.

In Fig. 7B, methane hydrate begins to form (${S}_{H}$ ~ 0.1) in the sand layer after approximately 380 kyr, coinciding with the sediment interval being buried at 73 mbsf. As modeling time progresses, hydrate saturation increases and reaches approximately ${S}_{H}$ ~ 0.5 of the pore space after 1200 kyr when the sediment interval is buried at 228 mbsf. In contrast to WR 313-H, the RTM predicts hydrate formation exclusively within the sand layer at U1325, with no hydrate observed in the surrounding muds.

In Fig. 7C, a comparison is drawn between the hydrate saturation predicted by the RTM and the hydrate saturations estimated from pore water chlorinity measurements at Site U1325. Both the RTM predictions and chlorinity measurements show that hydrate begins to form at 73 mbsf and reaches approximately ${S}_{H}$ ~ 0.5 near the BGHSZ. Both the pore water chlorinity and RTM results suggest that hydrate is only present within the sand layers and is generally absent in the surrounding muds.

## Discussion

The primary objective of this study is to explore how concentrated microbes in the sand layer gain access to solid organic matter in the adjacent muds, leading to organic carbon remineralization and the eventual formation of methane hydrate in the sand. The RTM successfully predicts methane hydrate saturations and distribution patterns that align with observed hydrate occurrences in two distinct marine environments. This suggests that the RTM is generally applicable across various geological settings, encompassing both passive and active continental margins. In these diverse settings, sediments are deposited at varying rates of sedimentation (ranging from 0.19 to 1 mm/year) and contain sand layers with substantially different thicknesses (ranging from a few cm to several meters).

The RTM also indicates that DOC is generated at relatively early times at both Sites, occurring at < 100 kyr at WR 313-H and < 400 kyr at U1325 (Figs. 5A, 7A). This observation suggests that microbial activity is most intense at shallow depths within the sediment column, well above the depth where significant methane hydrate accumulations occur. The hydrolysis of POC leads to the formation of DOC in the fine-grained sediment intervals. Subsequently, DOC diffuses into the sand layer, driven by a concentration gradient, and is effectively consumed there due to the presence of a large number of fermenting and methanogenic microbes. As a consequence, the concentration of DOC remains near zero throughout the modeled time interval in the sand layer (Figs. 5A, 7A). As POC is gradually depleted, the generation of DOC and the resulting microbial activity become progressively less intense.

**Figure 8:**
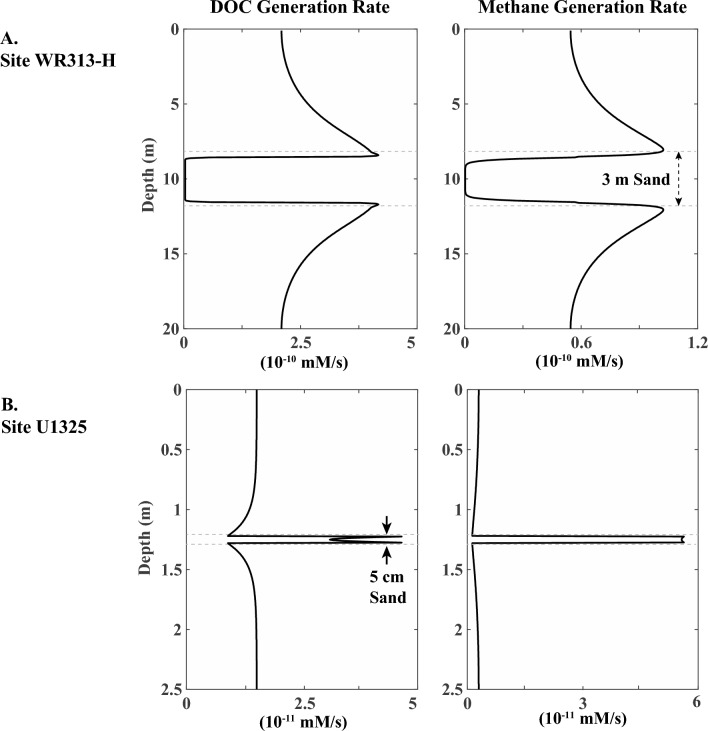
Results of reactive transport modeling (RTM) showing concentrated microbial activity at the sand–mud interface in both studied locations. (**A**) Modeled DOC generation rate and methane generation rate in a 20 m-thick mud-sand sediment interval buried at 100 mbsf at WR313-H, where a 3 m-thick sand layer is at the center of the interval surrounded by fine-grained muds. (**B**) Modeled DOC generation rate and methane generation rate in a 2.5 m-thick mud-sand sediment interval buried at 100 mbsf at Site U1325. A 5 cm-thick thin sand is at the center of the interval surrounded by mud.

The RTM predicts that the generation of DOC and methane is predominantly located near the boundary between the sand layer and the surrounding mud. This is where diffusion driven by concentration gradients transports solutes to the coarse-grained bed that harbors the highest microbe population (Fig. 8). This model prediction is consistent with the suggestion that subseafloor microbial activity tends to concentrate near lithological boundaries^51,52^. Additionally, it provides an explanation for the observation that the highest methane hydrate saturations occur near the top and base of the sand layer (Fig. 5B).

Microbial activity peaks during the early stages after burial at shallow depths (Fig. 5A,B). This results from the decrease in the available POC with time due to microbial consumption and associated reactions. Although microbial activity is concentrated at early times and shallow depths, methane hydrate keeps forming in the sand layers at greater depths. This is attributed to the diffusion of DOC and methane into the sand bed from the surrounding fine-grained muds. At Site WR313-H (Fig. 8A), methane hydrates initially form both above and below the sand bed, but as time progresses, they dissolve resulting in thickening of HFZs over time. The dissolution of methane hydrates in the fine-grained muds occurs due to methane diffusing out of the mud intervals, which progressively decreases the local methane concentration, and due to the increase in methane solubility with burial depth (and hence with time). In contrast, hydrates do not form in the fine-grained muds at Site U1325 (Fig. 8B), where the methane concentration never reaches the point of methane solubility which is required for methane hydrate formation. It appears that the conditions at Site U1325 are more favorable to methane diffusing away from fine-grained layers, which agrees with the LWD measurements and observations in the recovered core.

The primary objective of our study is to explore how concentrated microbes in a coarse-grained sand access solid organic matter in the adjacent muds even if there is no transport of methane by advective pore water flow. Consequently, we assumed no advective transport of methane by pore water flow between the mud and sand layers. Also, our model does not address deeper processes that occur near or below the GHSZ. The first reason is that the degradation of organic carbon primarily takes place at early times or shallow depths, and its intensity diminishes with increasing depth (Figs. 5A,B, 7B) while methane hydrate continues to form in the sand layer at greater depths due to diffusion of DOC and methane from the surrounding muds. Additionally, as sediment is buried below the base of the GHSZ, hydrate dissociates to water and free gas, leading to concentrated hydrate if methane is recycled. For the purpose of our study, we do not consider the processes of hydrate dissociation or methane recycling at the GHSZ.

## Summary of conclusions

We have developed an RTM that enhances the representation of microbially-driven POC degradation processes in marine methane hydrate systems. This marks a significant step toward RTMs that include explicit consideration of microbes. Our study demonstrates that the RTM effectively reproduces hydrate occurrences in two distinct geological environments: the northern Gulf of Mexico and the East Pacific Cascadia Margin. Notably, our results suggest that the majority of microbial activity is concentrated at relatively early times or shallow depths. Furthermore, our results highlight that microbes can degrade POC even if they are physically separated from the POC. This occurs as extracellular enzymes and DOC diffuse between sand layers and surrounding fine-grained muds. The generation of DOC and methane is concentrated at the lithological boundaries, where concentration gradients transport solutes to intervals that contain the most microbes. These model outcomes give biogeochemists and geologists an additional mechanism investigating the process of organic carbon degradation and methanogenesis: methane hydrates can form in coarse-grained layers from methane diffusing from adjacent fine-grained intervals. Our findings offer important perspectives to guide future sampling and measurements in offshore drilling expeditions, where the model we propose can be tested in different subseafloor environments.

### Supplementary Information

Supplementary Information.

## Data Availability

The datasets generated during the current study are available from the corresponding author on reasonable request.
