# Supplementary material for: Reactive transport modeling of organic carbon degradation in marine methane hydrate systems
Source: Sci Rep. 2024 Feb 3;14:2837. doi: 10.1038/s41598-024-52957-w (PMC10838294; doi:10.1038/s41598-024-52957-w)
Supplement: Supplementary file 1 — Supplementary Information. [file 41598_2024_52957_MOESM1_ESM.docx]

**Supplement**

1. **Time-dependent reactive transport model for microbial processes in marine methane hydrate systems**

**Figure S1.** (**A**) Unit volume of bulk sediment with no methane hydrate. Pore space occupies a fraction $\phi$ and sediment grains occupy ($1-\phi$) of the unit bulk volume. The sediment grains contain a mass fraction $\alpha$ of particulate organic carbon (POC) that is available for microbial processes. (**B**) Unit volume of bulk sediment with methane hydrate. Hydrate occupies a fraction $h\phi$ and pore water occupies $(1-h)\phi$ of the unit bulk volume. Densities of pore water, hydrate, and sediment grains are $\rho_{f}$, $\rho_{h}$, and $\rho_{s}$, respectively.

- 1. **Notation**

Solute concentrations of high-molecular-weight dissolved organic carbon $H$ (HMW-DOC), low-molecular-weight dissolved organic carbon $L$ (LWM-DOC), extracellular enzymes $E$, and methane $c_{m}$ are expressed as molar mass per unit volume of pore fluid ^1,23^. Solid terms are dimensionless; sedimentary particulate organic carbon (POC) $\alpha$ is in weight fraction of bulk sediment, and hydrate $h$ is in volume fraction of pore space (Fig. S1). The microbial reaction rate constants for POC hydrolysis are $K_{o}$ and $K_{eh}$, the reaction rate constant for HMW-DOC fermentation is $K_{fm}$ and for methanogenesis is $K_{m}$. The diffusion coefficients of solute HMW-DOC, LMW-DOC, Enzymes, and methane in the sediment pore water are $D_{Hs}$, $D_{Ls}$, $D_{Es}$, and $D_{ms}$, respectively. The equilibrium methane concentration is written as solubility $s$. Time- and depth-dependent terms are explicitly listed in Table S1 as functions of $t$ and $z$ to distinguish them from constant terms. In the Lagrangian framework used here (described in section 1.3) depth $z$ is referred to the top of the modeled sediment interval.

**Table S1.** List of symbols, acronyms and values observed in two studied drill sites. GHSZ = gas hydrate stability zone; mbsf = meters below seafloor.

| Symbol | Definition | Value at WR313-H | Value at U1325 | Units |
| --- | --- | --- | --- | --- |
| $B_{Fs}$ | Number of fermenting microbial cells in a cubic m of pore water in sand^4^ | 3$\cdot$10^12^ | 3$\cdot$10^12^ | - |
| $B_{Fm}$ | Number of fermenting microbial cells in a cubic m of pore water in mud^4^ | 1$\cdot$10^15^ | 1$\cdot$10^15^ | - |
| $B_{Ms}$ | Number of methanogen cells in a cubic m of pore water in sand^4^ | 3$\cdot$10^12^ | 3$\cdot$10^12^ | - |
| $B_{Mm}$ | Number of methanogen cells in a cubic m of pore water in mud^4^ | 1$\cdot$10^15^ | 1$\cdot$10^15^ | - |
| $c_{h}$ | Methane concentration in gas hydrate^3^ | 7456 | 7456 | mM |
| $c_{m}(t,z)$ | Methane concentration in pore water | - | - | mM |
| $D_{Es}$ | Diffusion coefficient of extracellular enzymes in pore water^5^ | 1.9$\cdot$10^-11^ | 2.4$\cdot$10^-11^ | m^2^$\cdot$s^-1^ |
| $D_{Hs}$ | Diffusion coefficient of HMW-DOC in pore water^6,7^ | 5.9$\cdot$10^-13^ | 7.5$\cdot$10^-13^ | m^2^$\cdot$s^-1^ |
| $D_{Ls}$ | Diffusion coefficient of LMW-DOC in pore water^6,7^ | 8.7$\cdot$10^-12^ | 1.1$\cdot$10^-11^ | m^2^$\cdot$s^-1^ |
| $D_{ms}$ | Diffusion coefficient of methane in pore water^8^ | 3.2$\cdot$10^-10^ | 4$\cdot$10^-10^ | m^2^$\cdot$s^-1^ |
| $E(t,z)$ | Extracellular enzymes concentration in pore water | - | - | mM |
| $H(t,z)$ | HMW-DOC concentration in pore water | - | - | mM |
| $h(t,z)$ | Hydrate saturation (volume fraction of pore space) | - | - | - |
| $K_{eh}$ | POC hydrolysis rate constant driven by extracellular enzymes | - | - | mM^-1^$\cdot$s^-1^ |
| $K_{ed}$ | Extracellular enzymes decay rate constant | - | - | s^-1^ |
| $K_{fm}$ | Fermentation rate constant per microbial cell | - | - | s^-1^ |
| $K_{h}$ | Hydrate formation/dissolution rate constant | - | - | s^-1^ |
| $K_{m}$ | Methanogenesis rate constant per microbial cell | - | - | s^-1^ |
| $K_{o}$ | Background POC hydrolysis rate constant | - | - | s^-1^ |
| $L(t,z)$ | LMW-DOC concentration in pore water | - | - | mM |
| $Q_{h}(t,z)$ | Gas hydrate formation rate | - | - | s^-1^ |
| $s(z)$ | Methane solubility in pore water | - | - | mM |
| $v_{s}$ | Sedimentation rate^3,9^ | 3.17$\cdot$10^-11^ | 6.2$\cdot$10^-12^ | m$\cdot$s^-1^ |
| $z_{BGHSZ}$ | Depth to base of GHSZ^3,10^ | 900 | 241 | mbsf |
| $\alpha(t,z)$ | POC content in weight fraction of sediment grains^3^ | 0.5 | 0.5 | wt. % |
| $\beta$ | Ratio of POC background hydrolysis rate constant to enzyme-driven hydrolysis rate | 0.01 | 0.01 | - |
| $\varepsilon$ | Ratio of generation rate of extracellular enzymes to LMW-DOC generation rate | 0.01 | 0.01 | - |
| $\lambda$ | Half-life time of extracellular enzymes^11,12^ | 10 | 10 | kyr |
| $\rho_{s}$ | Density of sediment grains^3^ | 2750 | 2750 | kg$\cdot$m^-3^ |
| $\rho_{f}$ | Density of pore water | 1000 | 1000 | kg$\cdot$m^-3^ |
| $\rho_{h}$ | Density of gas hydrate | 910 | 910 | kg$\cdot$m^-3^ |
| $\phi$ | Sediment porosity^3,9^ | 0.35 | 0.45 | - |

- 1. **Local mass balance equations**
     1. *Conversion between POC and HMW-DOC*

In Fig. S1, mass fraction of POC of sediment grains is $\alpha$. Thus, the mass of POC per unit bulk volume (sediment grains and pore water), in kg$\cdot$m^-3^, is

$\alpha\cdot\rho_{s}\cdot\left( 1-\phi\right)$.

( 1 )

As C has a molar mass of 12 grams, the molar mass of POC per unit bulk volume, in mole$\cdot$m^-3^ or mM, is

$\frac{\alpha\cdot\rho_{s}\cdot\left( 1-\phi\right)}{0.012}$.

( 2 )

If all POC is converted to HMW-DOC, Equation (2) gives the molar mass of HMW-DOC per unit bulk volume. As HMW-DOC is dissolved in pore water, the concentration of HMW-DOC per unit volume of pore water is the same as Equation (2) divided by the porosity (volume of pore water in the unit bulk volume) as in

$\frac{\rho_{s}\cdot(1-\phi)}{0.012\cdot(1-h)\phi}\alpha$,

( 3 )

where $h$ is the methane hydrate saturation ($h=0$ if hydrate is not present). The conversion factor between POC and HMW-DOC is

$$\frac{\rho_{s}\cdot(1-\phi)}{0.012\cdot(1-h)\phi}.$$

( 4 )

- - 1. *Sedimentary POC content*

The mass of POC in weight fraction of sediment grains is $\alpha$. Mass conservation for POC decomposition is expressed by equating the time derivative of the POC mass with the sum of transport due to sediment burial and reaction terms. As the depth frame of reference is fixed to the top of the sediment interval, the sediment burial and pore fluid velocity are both zero in the Lagrangian framework. Therefore, POC mass conservation is only affected by reaction terms, which are background hydrolysis with a rate constant $K_{o}$ (s^–1^) and extracellular enzymes-driven hydrolysis with a rate constant $K_{eh}$ (mM^–1^$\cdot$s^–1^):

$\frac{\partial}{\partial t}\alpha=-\left( K_{eh}E+K_{o} \right)\cdot\alpha$.

( 5 )

Note that there is an mM^-1^ in the units of $K_{eh}$ as the POC hydrolysis is controlled by the concentration of enzymes $E$ (mM) in pore water.

- - 1. *Mass of HMW-DOC*

Mass conservation for HMW-DOC in the pore water is expressed by equating the time derivative of HMW-DOC concentration $H$ to the sum of a diffusion transport term (advection is zero in the Lagrangian framework) with a coefficient $D_{H}$ (m^2^ $\cdot$s^-1^) plus the HMW-DOC generation from POC hydrolysis and minus the consumption of HMW-DOC from fermentation:

$\frac{\partial}{\partial t}H=\frac{\partial}{\partial z}D_{H}\frac{\partial H}{\partial z}+\frac{\rho_{s}\cdot(1-\phi)}{0.012\cdot\left( 1-h \right)\phi}\cdot\left( K_{eh}E+K_{o} \right)\cdot\alpha-B_{fm}K_{fm}H$,

( 6 )

where $B_{fm}$ is the number of cells in a m^3^ of pore fluid and $K_{fm}$ is the microbial fermentation rate constant per cell (s^–1^).

- - 1. *Mass of Extracellular enzymes*

Mass conservation for extracellular enzymes in the pore water is expressed by equating the time derivative of extracellular enzyme concentration $E$ with the sum of a diffusion transport term with a coefficient $D_{E}$ (m^2^$\cdot$s^-1^) plus enzyme generation from HMW-DOC fermentation at a small fraction $\varepsilon\ll1$ and minus enzyme decay with a rate constant $K_{ed}$ (s^-1^):

$\frac{\partial}{\partial t}E= \frac{\partial}{\partial z}D_{E}\frac{\partial E}{\partial z}+{\varepsilon\cdot B}_{fm}K_{fm}H-K_{ed}E$,

( 7 )

where $K_{ed}$ (s^–1^) is calculated from a half-life time of enzymes $\lambda$ (kyr):

$K_{ed}=\frac{log(2)}{\lambda(10000\times365\times24\times3600)}$.

( 8 )

- - 1. *Mass of LMW-DOC*

Mass conservation for LMW-DOC in the pore water is expressed by equating the time derivative of LMW-DOC concentration $L$ with the sum of a diffusion transport term with a coefficient $D_{L}$ (m^2^$\cdot$s^-1^) plus the generation of LMW-DOC from HMW-DOC fermentation at a fraction $(1-\varepsilon)$ near unity and minus the LMW-DOC consumption due to microbial methanogenesis at a rate constant $K_{m}$ (s^–1^):

$\frac{\partial}{\partial t}L= \frac{\partial}{\partial z}D_{L}\frac{\partial L}{\partial z}+{(1-\varepsilon)\cdot B}_{fm}K_{fm}H-B_{m}K_{m}L$,

( 9 )

where $B_{m}$ is the number of methanogen cells in a m^3^ of pore water and $K_{m}$ (s^–1^) is the microbial methanogenesis rate constant per cell, which is assumed to be equal to the fermentation rate constant $K_{fm}$.

**Figure S2.** (**A**) In a unit volume of sediment, the volume of pore space $\phi$ contains a fraction $(1-h)$ of pore water with a dissolved methane concentration ${(c}_{m}+\Delta c_{m})$ in mole$\cdot$m^-3^ or mM and a fraction of hydrate $h$. (**B**) Conversion of the excess dissolved methane $\Delta c_{m}$ creates an additional volume of hydrate $\Delta h$. The total mass of methane in the pore space remains the same in A and B.

- - 1. *Conversion between dissolved methane and hydrate*

In Fig. S2A, the total molar mass of methane in pore space is the sum of molar mass of methane in pore water and methane in hydrate, which gives:

${(c}_{m}+\Delta c_{m})\cdot(1-h)+c_{h}\cdot h$,

( 10 )

where $\Delta c_{m}$ is the methane concentration above solubility $s$ and $c_{h}$is the molar mass of methane per unit volume of hydrate, in mole$\cdot$m^-3^ or mM. As the mass concentration of methane in hydrate is 119.3 kg$\cdot$m^-3^ ^3^ and the molar mass of methane is 16 g, $c_{h}$ is

$c_{h}=\frac{119.3}{0.016}$.

( 11 )

Similarly, in Fig. S2B, the total molar mass of methane in pore space is

$c_{m}\cdot\left( 1-h-\Delta h \right)+c_{h}\cdot\left( h+\Delta h \right)$,

( 12 )

where $\Delta h$ is the amount of hydrate that forms from the excess methane concentration $\Delta c_{m}$. As the total molar mass of methane in pore space is the same in Fig. S2A and Fig. S2B, we have:

${(c}_{m}+\Delta c_{m})\cdot\left( 1-h \right)+c_{h}\cdot h=c_{m}\cdot\left( 1-h-\Delta h \right)+c_{h}\cdot\left( h+\Delta h \right)$,

( 13 )

which results in

$$\Delta c_{m}= \frac{c_{h}-c_{m}}{1-h}\cdot\Delta h$$

( 14 )

or

$\Delta h= \frac{1-h}{c_{h}-c_{m}}\cdot\Delta c_{m}$.

( 15 )

The factors $\frac{c_{h}-c_{m}}{1-h}$ and $\frac{1-h}{c_{h}-c_{m}}$ are conversion factors between methane in solution $c_{m}$ and hydrate $h$.

If the solubility of methane in pore water is $s$ (mM or mole$\cdot$m^3^), when $c_{m}>s$ the excess methane concentration $\Delta c_{m}= c_{m}-s$ will form an amount of hydrate $\Delta h$ within the GHSZ that is given by:

$\Delta h= \frac{1-h}{c_{h}-s}\cdot\Delta c_{m}$.

( 16 )

If the methane concentration $c_{m}$ < $s$ and all hydrate $h$ dissolves back into methane and water following Equation (14), the created additional amount of dissolved methane in mole$\cdot$m^–3^ (or mM) is given by:

$\Delta c_{m}= {(c}_{h}-c_{m})\cdot h$.

( 17 )

If the methane concentration $c_{m}$ < $s$, and only a fraction of the hydrate present dissolves to a concentration level of $s$, the amount of dissolving hydrate is given by:

$$\Delta h= \frac{1-h}{c_{h}-c_{m}}\cdot\left( s-c_{m} \right).$$

( 18 )

- - 1. *Methane hydrate volume fraction*

Mass conservation of solid methane hydrate is expressed as:

$\frac{\partial}{\partial t}h= Q_{h}$,

( 19 )

where $Q_{h}$ is the formation rate of hydrate if $c_{m}$ > $s$, or the dissolution of hydrate otherwise. $Q_{h}$ is calculated by:

$$Q_{h}= \left\{ \begin{aligned} 0, if c_{m}\leq s &and h=0; \\ K_{h}\cdot\frac{1-h}{c_{h}-s}\left( c_{m}-s \right), otherwise, \end{aligned} \right.$$

( 20 )

where $K_{h}$ (s^-1^) is the hydrate formation/dissolution rate constant and is assumed to be much greater than all other microbial reaction rates to account for the fast kinetics of hydrate formation/dissolution. The sign of $Q_{h}$will be positive if $c_{m}$ > $s$ and negative if $c_{m}$ < $s$ and hydrate is present ($h$ > $0$).

- - 1. *Mass of dissolved methane*

Mass conservation of methane in pore water is expressed by equating the time derivative of methane concentration $c_{m}$ with the sum of diffusion transport with a coefficient $D_{m}$(m^2^$\cdot$s^-1^) plus methane generation from methanogenesis that consumes LWM-DOC, and minus conversion between methane in solution and hydrate:

$\frac{\partial}{\partial t}c_{m}=\frac{\partial}{\partial z}D_{m}\frac{\partial c_{m}}{\partial z}+{\frac{1}{2}B}_{m}K_{m}L-\frac{c_{h}-c_{m}}{1-h}\cdot Q_{h}$,

( 21 )

where the factor of 1/2 accounts for only half of the carbon in LMW-DOC being converted to methane in the methanogenesis process.

- 1. **Finite difference solution**

This section describes the finite difference method used to solve for the sedimentary POC content ($\alpha$), the dissolved concentrations of HMW-DOC ($H$), extracellular enzymes ($E$), LMW-DOC ($L$), methane concentration ($c_{m}$), and the solid hydrate saturation ($h$) in the reactive transport equations, (i.e., Equation (5), (6), (7), (9), (19), and (21)). The modeling domain is discretized in a $m\times n$ matrix for depths $z=[ z_{1}, z_{2},\ldots, z_{m}]$ and times $t=[ t_{1}, t_{2}, \ldots, t_{n}]$. Depths $z_{1}$ and $z_{m}$ refer to the top and base of the sediment interval and times $t_{1}$ and $t_{n}$ refer to the initial and end time, respectively. Solutions are calculated for the model depth interval at time $t_{i+1}$ based on the solution at the previous time $t_{i}$.

In the Lagrangian framework used, the depth coordinate is fixed to the top of the moving sediment interval at an initial time $t_{1}$, from the seafloor to greater depths. At the initial time $t_{1}$, the model starts with a small amount of POC that is only present in the fine-grained mud interval, concentrated microbial cells in the sand layer, and zero solutes and hydrate throughout the sediment interval. Because there is no relative movement between the sediment interval and the Lagrangian coordinate, advection terms are zero and are not included in the reactive transport equations, (i.e., Equation (5), (6), (7), (9), (19), and (21)). We impose zero-gradient Neumann boundary conditions (BCs) at the top ($z_{1}$) and bottom ($z_{m}$) of the sediment interval, meaning that no flow of carbon is allowed into or out of the sediment interval and that carbon mass is conserved.

We apply a backward Euler implicit method with a procedure that takes three fractional steps at each time step $\Delta t$, and is second-order accurate^13^. The first fractional step accounts for solute diffusion (HMW-DOC, enzymes, LMW-DOC, and methane) over a half time step $\Delta t/2$. The second fractional step accounts for the reaction terms of POC hydrolysis, microbial fermentation and methanogenesis, enzymes generation and decay, and hydrate formation/dissolution over a time step $\Delta t$. The third fractional step accounts for solute diffusion over the other half time step $\Delta t/2$. Details of the solution are shown below.

- - 1. *Fractional step 1: diffusion terms (half time step* $\Delta t/2$*)*

Using finite difference implicit method, the solution for solute diffusion over a half time step $\Delta t/2$ in Equation (6), (7), (9), and (21) can be written as

$\frac{\underline{X}_{i+\frac{1}{2}}-\underline{X}_{i}}{{\Delta t}/2}=\frac{D_{X}}{\Delta z^{2}}\underline{\underline{M}} \underline{X}_{i+\frac{1}{2}}$ ,

( 22 )

where $\underline{X}$ is a vector that contains the solute concentration over the model depth interval for HMW-DOC ($H$), extracellular enzymes ($E$), LMW-DOC ($L$), and methane ($c_{m}$); $\underline{X}_{i}$ and $\underline{X}_{i+\frac{1}{2}}$ are the solute concentrations of $\underline{X}$ at time $t_{i}$ and $t_{i+\frac{1}{2}}=t_{i}+\frac{\Delta t}{2}$; $D_{X}$ is the diffusion coefficient of the solute in sediment; and $\underline{\underline{M}}$ is a tridiagonal $m\times m$ matrix (we denote vectors with a single underline and matrices with a double underline):

$\underline{\underline{M}}=\left[ \begin{matrix} -2 & 1 & & & & \\ 1 & -2 & 1 & & & \\ & 1 & -2 & 1 & & \\ & & \ddots& \ddots& \ddots& \\ & & & 1 & -2 & 1 \\ & & & & 1 & -2 \end{matrix} \right]$.

( 23 )

Multiplying both sides of Equation (22) by $\frac{\Delta t}{2}$ and rearranging terms gives a linear implicit equation for $\underline{X}$ at time $t_{i+\frac{1}{2}}=t_{i}+\frac{\Delta t}{2}$:

$\underline{\underline{G}} \underline{X}_{i+\frac{1}{2}}= \underline{X}_{i}$,

( 24 )

with

$\underline{\underline{G}}=\underline{\underline{I}}-\eta D_{X}\cdot\underline{\underline{M}}$,

( 25 )

$\eta=\frac{\Delta t}{2\Delta z^{2}}$,

( 26 )

where $\underline{\underline{I}}$ is an $m\times m$ identity matrix, and $\underline{\underline{G}}$ is a $m\times m$ tridiagonal matrix. To impose no flux BCs at the top and base of the sediment interval, the first and the last rows in $\underline{\underline{G}}$ are modified to enforce a zero gradient as in

$\underline{\underline{G}}=\left[ \begin{matrix} -\frac{3}{2\Delta z} & \frac{2}{\Delta z} & -\frac{1}{2\Delta z} & & & \\ -\eta D_{X} & 1+2\eta D_{X} & -\eta D_{X} & & & \\ & -\eta D_{X} & 1+2\eta D_{X} & -\eta D_{X} & & \\ & & \ddots& \ddots& \ddots& \\ & & & -\eta D_{X} & 1+2\eta D_{X} & -\eta D_{X} \\ & & & \frac{1}{2\Delta z} & -\frac{2}{\Delta z} & \frac{3}{2\Delta z} \end{matrix} \right]$.

( 27 )

- - 1. *Fractional step 2: reaction terms (full time step* $\Delta t$*)*
       1. *Sedimentary POC hydrolysis*

Following Equation (5), for a time step $\Delta t$, the mass of POC at time $t_{i+1}$ is calculated based on the value at time $t_{i}$,

$\alpha_{i+1}=\alpha_{i}(1-Q_{\alpha})$,

( 28 )

where $Q_{\alpha}$ is dimensionless, expressed by the background hydrolysis and enzymes-driven hydrolysis:

$Q_{\alpha}=e^{-(K_{eh}E+K_{o})\Delta t}$.

( 29 )

- - - 1. *HMW-DOC generation and fermentation*

Following Equation (28), the mass of HMW-DOC generated from POC hydrolysis during a time step $\Delta t$ is calculated as

$\frac{\rho_{s} (1-\phi)}{0.012\cdot\left( 1-h \right)\phi}\cdot\alpha_{i}Q_{\alpha}$,

( 30 )

where the factor $\frac{\rho_{s} (1-\phi)}{0.012\cdot\left( 1-h \right)\phi}$ is applied to convert solid POC to the concentration of carbon (moles) in pore water (Fig. S1).

The concentration of HMW-DOC at time $t_{i+1}$ due to fermentation is calculated based on the value at previous time as

$H_{i+1}=H_{i}(1-Q_{H})$,

( 31 )

where the term $Q_{H}$ is

$Q_{H}=e^{-{(B}_{fm}K_{fm})\Delta t}$.

( 32 )

The concentration of HMW-DOC after a time step $\Delta t$ of POC hydrolysis is $H_{i+\frac{1}{2}}^{*}$as in

$H_{i+\frac{1}{2}}^{*}=\frac{\rho_{s} (1-\phi)}{0.012\cdot\left( 1-h \right)\phi}\alpha_{i}Q_{\alpha}-{H_{i+\frac{1}{2}}Q}_{H}$.

( 33 )

- - - 1. *Extracellular enzymes generation and decay*

The generation of extracellular enzymes from HMW-DOC fermentation over a time step $\Delta t$ is

$$\varepsilon\cdot{H_{i+\frac{1}{2}}Q}_{H}$$

( 34 )

and the concentration of enzymes that decay during a time step $\Delta t$ is

${E\cdot K}_{ed}\cdot\Delta t$.

( 35 )

Therefore, the concentration of enzymes after a time step $\Delta t$ of HMW-DOC fermentation and decay is $E_{i+\frac{1}{2}}^{*}$as in

$E_{i+\frac{1}{2}}^{*}=\varepsilon\cdot{H_{i+\frac{1}{2}}Q}_{H}-{E_{i+\frac{1}{2}}\cdot K}_{ed}\cdot\Delta t$.

( 36 )

- - - 1. *LMW-DOC generation and methanogenesis*

The generation of LMW-DOC from HMW-DOC fermentation over a time step $\Delta t$ is

$(1-\varepsilon{)H_{i+\frac{1}{2}}Q}_{H}$.

( 37 )

Methanogenesis consumes LMW-DOC, so that its concentration after a time step $\Delta t$ equals

$L_{i+1}=L_{i}(1-Q_{L})$,

( 38 )

where the term $Q_{L}$ is

$Q_{L}=e^{-{(B}_{m}K_{m})\Delta t}$.

( 39 )

Therefore, the concentration of LMW-DOC after a time step $\Delta t$ of HMW-DOC fermentation and methanogenesis is $L_{i+\frac{1}{2}}^{*}$as in

$L_{i+\frac{1}{2}}^{*}=\left( 1-\varepsilon\right)H_{i+\frac{1}{2}}Q_{H}-L_{i+\frac{1}{2}}Q_{L}$.

( 40 )

- - - 1. *Methane concentration and hydrate saturation*

As methanogenesis consumes one mole of carbon to generate half a mole of methane, the concentration of methane after a time step $\Delta t$ of LMW-DOC consumption is ${c_{m}}_{i+\frac{1}{2}}^{*}$, and thus:

${c_{m}}_{i+\frac{1}{2}}^{*}={c_{m}}_{i+\frac{1}{2}}+{\frac{1}{2}L}_{i+\frac{1}{2}}Q_{L}$.

( 41 )

To account for hydrate formation/dissolution, the solutions for methane concentration ${c_{m}}_{i+\frac{1}{2}}^{**}$ and hydrate saturation $h_{i+1}$ after a time step $\Delta t$ are as follows:

- If ${c_{m}}_{i+\frac{1}{2}}^{*}>s$, enough hydrate forms to lower the methane concentration to the solubility value. From Equation (15), we have

$${c_{m}}_{i+\frac{1}{2}}^{**}=s$$

( 42 )

$$h_{i+1}=h_{i}+\frac{1-h_{i}}{c_{h}-s} ({c_{m}}_{i+\frac{1}{2}}^{*}-s)$$

( 43 )

- If ${c_{m}}_{i+\frac{1}{2}}^{*}\leq s$ and $h_{i}=0$, no hydrate forms or dissolves:

$${c_{m}}_{i+\frac{1}{2}}^{**}={c_{m}}_{i+\frac{1}{2}}^{*}$$

( 44 )

$$h_{i+1}=0$$

( 45 )

- If ${c_{m}}_{i+\frac{1}{2}}^{*}<s$ and $h_{i}>0$, we calculate the amount of methane created if all hydrate dissolves. From Equation (17), we have

$${c_{m}}_{i+\frac{1}{2}}^{**}={c_{m}}_{i+\frac{1}{2}}^{*}+\left( c_{h}-{c_{m}}_{i+\frac{1}{2}}^{*} \right)h_{i}$$

( 46 )

$$h_{i+1}=0$$

( 47 )

If the calculated ${c_{m}}_{i+\frac{1}{2}}^{**}\leq s$, hydrate dissolution is complete. If the calculated ${c_{m}}_{i+\frac{1}{2}}^{**}>s$, only a portion of hydrate dissolves to increase methane concentration and equals the local solubility, we then have

$${c_{m}}_{i+\frac{1}{2}}^{**}=s$$

( 48 )

$h_{i+1}=h_{i}+\frac{1-h_{i}}{c_{h}-{c_{m}}_{i+\frac{1}{2}}^{*}} ({c_{m}}_{i+\frac{1}{2}}^{*}-s)$

( 49 )

- - 1. *Fractional step 3: diffusion terms (half time step* $\Delta t/2$*)*

The third fractional step accounts for diffusion with a half time step $\Delta t/2$ by solving again Equation (24), and thus:

$\underline{\underline{G}} \underline{X}_{i+1}=\underline{X}_{i+\frac{1}{2}}^{*}$,

( 50 )

where $\underline{X}_{i+\frac{1}{2}}^{*}$ contains the concentrations of dissolved components obtained in fractional step 2 (section 1.3.2), which are $H_{i+\frac{1}{2}}^{*}$, $E_{i+\frac{1}{2}}^{*}$, $L_{i+\frac{1}{2}}^{*}$, and ${c_{m}}_{i+\frac{1}{2}}^{**}$.

1. **DOC concentration in sediment pore water from scientific ocean drilling sites**

**Table S2.** Dissolved organic carbon concentrations in interstitial water from 812 measurements at 26 sites sampled by Scientific Ocean Drilling (* from the IODP web database)

| Location | Site | Maximum DOC (mM) | Minimum DOC (mM) | Total measurements |
| --- | --- | --- | --- | --- |
| Baja California margin^14^ | 474 | 16.98 | 2.58 | 13 |
| Guaymas Basin Slope^14^ | 479 | 15.65 | 1.67 | 11 |
| Orca Basin^15^ | 618 | 18.73 | 5.24 | 11 |
| Pigmy Basin^15^ | 619 | 9.99 | 4.66 | 20 |
| Mississippi Fan^15^ | 623 | 3.58 | 1.91 | 8 |
| Tyrrhenian Sea^16,17^ | 651 | 2.45 | 0.37 | 9 |
| Tyrrhenian Sea^16,17^ | 653 | 8.34 | 0.26 | 13 |
| Peru Margin^16,17^ | 681 | 4.12 | 0.51 | 16 |
| Middle Valley^18^ | 855 | 1.62 | 0.17 | 27 |
| Middle Valley^18^ | 856 | 0.87 | 0.01 | 26 |
| Middle Valley^18^ | 857 | 6.77 | 0.18 | 35 |
| Middle Valley^18^ | 858 | 7.13 | 0.01 | 55 |
| Blake Ridge^19^ | 997 | 93.4 | 1.36 | 23 |
| Hydrate Ridge^20^ | 1244 | 19.19 | 1.91 | 17 |
| Peru Margin^21^ | 1225 | 1.53 | 0.19 | 26 |
| Peru Margin^21^ | 1226 | 0.72 | 0.32 | 31 |
| Peru Margin^21^ | 1227 | 2.92 | 0.69 | 53 |
| Peru Margin^21^ | 1228 | 1.75 | 0.35 | 47 |
| Peru Margin^21^ | 1229 | 1.6 | 0.48 | 47 |
| Peru Margin^21^ | 1230 | 23.58 | 1.66 | 92 |
| Peru Margin^21^ | 1231 | 1.7 | 0.33 | 42 |
| Cascadia Margin^22^ | 1329 | 5.1 | 0.4 | 45 |
| Walvis Basin^22^ | 1082 | 9 | 0.5 | 45 |
| Juan de Fuca Ridge* | 1301 | 19.3 | 0.31 | 34 |
| Guatemala Basin on the Cocos plate* | 1256 | 2.75 | 0.04 | 97 |
| North Atlantic Ocean^23^ | 603 | 10.5 | 2.8 | 14 |

**References**

1. Davie, M. K. & Buffett, B. A. A numerical model for the formation of gas hydrate below the seafloor. *J Geophys Res Solid Earth* **106**, 497–514 (2001).

2. Davie, M. K. & Buffett, B. A. A steady state model for marine hydrate formation: Constraints on methane supply from pore water sulfate profiles. *J Geophys Res Solid Earth* **108**, 1–13 (2003).

3. Malinverno, A. Marine gas hydrates in thin sand layers that soak up microbial methane. *Earth Planet Sci Lett* **292**, 399–408 (2010).

4. Park, J. & Santamarina, J. C. The critical role of pore size on depth-dependent microbial cell counts in sediments. *Sci Rep* **10**, (2020).

5. Traving, S. J., Thygesen, U. H., Riemann, L. & Stedmon, C. A. A model of extracellular enzymes in free-living microbes: Which strategy pays off? *Appl Environ Microbiol* **81**, 7385–7393 (2015).

6. Burdige, D. J., Alperin, M. J., Homstead, J. & Martens, C. S. The Role of Benthic Fluxes of Dissolved Organic Carbon in Oceanic and Sedimentary Carbon Cycling. *Geophys Res Lett* **19**, 1851–1854 (1992).

7. Rossel, P. E., Bienhold, C., Hehemann, L., Dittmar, T. & Boetius, A. Molecular Composition of Dissolved Organic Matter in Sediment Porewater of the Arctic Deep-Sea Observatory HAUSGARTEN (Fram Strait). *Front Mar Sci* **7**, (2020).

8. Iversen, N. & Jorgensen, B. B. Diffusion Coefficients of Sulfate and Methane in Marine Sediments: Influence of Porosity. *Geochim Cosmochim Acta* **57**, 571–578 (1993).

9. Cook, A. E. & Malinverno, A. Short migration of methane into a gas hydrate-bearing sand layer at Walker Ridge, Gulf of Mexico. *Geochemistry, Geophysics, Geosystems* **14**, 283–291 (2013).

10. Boswell, R. *et al.* Architecture of gas-hydrate-bearing sands from Walker Ridge 313, Green Canyon 955, and Alaminos Canyon 21: northern Deepwater Gulf of Mexico. *Mar Pet Geol* **34**, 134–149 (2012).

11. Steen, A. D. & Arnosti, C. Long lifetimes of β-glucosidase, leucine aminopeptidase, and phosphatase in Arctic seawater. *Mar Chem* **123**, 127–132 (2011).

12. Schmidt, J. M., Royalty, T. M., Lloyd, K. G. & Steen, A. D. Potential activities and long lifetimes of organic carbon-degrading extracellular enzymes in deep subsurface sediments of the Baltic Sea. *Front Microbiol* **12**, (2021).

13. LeVeque, R. J. *Finite difference methods for ordinary and partial differential equations steady-state and time-dependent problems*. (2007).

14. Michaelis, W., Mycke, B., Vogt, J., Schuetze, G. & Degens, E. T. *39. Organic geochemistry of interstitial waters, sites 474 and 479, LEG 64*. (1982).

15. Ishizuka, T., Ittekkot, V., Degens, E. T. & Kawahata, H. *43. Preliminary data on dissolved organic carbon and sugar in interstitial water from the Mississippi fan and Orca and Pigmy basins, Deep Sea Drilling Project LEG 96*. (1986).

16. Seifert, R., Emeis, K.-C., Michaelis, W. & Degens, E. T. *36. Amino acids and carbohydrates in sediments and interstitial waters from Site 681, LEG 112, Peru Continental Margin*. *Scientific Results* vol. 112 (1990).

17. Seifert, R. *et al.* *36. Geochemistry of labile organic matter in sediments and interstitial water recovered from Sites 651 and 653, ODP LEG 107 in the Tyrrhenian Sea*. *Scientific Results* vol. 107 (1990).

18. Ran, B. & Simoneit, B. R. T. *23. Dissolved Organic Carbon in Interstitial Waters from Sediments of Middle Valley, LEG 139*. *Scientific Results* vol. 139 (1994).

19. Egeberg, P. Kr. & Barth, T. Contribution of dissolved organic species to the carbon and energy budgets of hydrate bearing deep sea sediments (Ocean Drilling Program Site 997 Blake Ridge). *Chem Geol* **149**, 25–35 (1998).

20. Tréhu, A. M., Bohrmann, G., Rack, F. R. & Torres, M. E. *Proceedings of the ocean drilling program, Initial Reports Volume 204*. doi:10.2973/odp.proc.ir.204.2003 (2003).

21. Smith, D. C. *9. Data report: dissolved organic carbon in interstitial waters, equatorial Pacific and Peru Margin, ODP LEG 201*. vol. 201 http://www-odp.tamu.edu/ (2005).

22. Heuer, V. B., Pohlman, J. W., Torres, M. E., Elvert, M. & Hinrichs, K. U. The Stable Carbon Isotope Biogeochemistry of Acetate and Other Dissolved Carbon Species in Deep Subseafloor Sediments at The Northern Cascadia Margin. *Geochim Cosmochim Acta* **73**, 3323–3336 (2009).

23. Emeis, K.-C., Mycke, B., Richnow, H.-H., Spitzy, A. & Degens, E. T. *56. Organic carbon and nitrogen, sediment composition, and clay mineralogy of deep-sea drilling project Site 603, Western Atlantic Ocean*. (1987).
